# Alterations in Gene Expression during Incompatible Interaction between Amendoim Cavalo Common Bean and *Colletotrichum lindemuthianum*

**DOI:** 10.3390/plants13091245

**Published:** 2024-04-30

**Authors:** Maike Lovatto, Pedro Soares Vidigal Filho, Maria Celeste Gonçalves-Vidigal, Mariana Vaz Bisneta, Alexandre Catto Calvi, Thiago Alexandre Santana Gilio, Eduardo A. Nascimento, Maeli Melotto

**Affiliations:** 1 Departamento de Agronomia, Universidade Estadual de Maringá, Maringá, PR 87020-900, Brazil; 2 Instituto de Ciências Agrárias e Ambientais, Universidade Federal do Mato Grosso, Sinop, MT 78060-900, Brazil; 3 Department of Plant Sciences, University of California, Davis, CA 95616, USA

**Keywords:** candidate gene expression, common bean–anthracnose interaction, plant defense genes

## Abstract

Anthracnose, caused by the fungus *Colletotrichum lindemuthianum*, poses a significant and widespread threat to the common bean crop. The use of plant genetic resistance has proven to be the most effective strategy for managing anthracnose disease. The Amendoim Cavalo (AC) Andean cultivar has resistance against multiple races of *C. lindemuthianum*, which is conferred by the *Co-AC* gene. Fine mapping of this resistance gene to common bean chromosome Pv01 enabled the identification of *Phvul.001G244300*, *Phvul.001G244400*, and *Phvul.001G244500* candidate genes for further validation. In this study, the relative expression of *Co-AC* candidate genes was assessed, as well as other putative genes in the vicinity of this locus and known resistance genes, in the AC cultivar following inoculation with the race 73 of *C. lindemuthianum*. Gene expression analysis revealed significantly higher expression levels of *Phvul.001G244500*. Notably, *Phvul.001G244500* encodes a putative Basic Helix–Loop–Helix transcription factor, suggesting its involvement in the regulation of defense responses. Furthermore, a significant modulation of the expression of defense-related genes PR1a, PR1b, and PR2 was observed in a time-course experiment. These findings contribute to the development of improved strategies for breeding anthracnose-resistant common bean cultivars, thereby mitigating the impact of this pathogen on crop yields and ensuring sustainable bean production.

## 1. Introduction

The common bean (*Phaseolus vulgaris* L.) holds the distinction of being the most widely consumed legume in human diets [1]. Known for its affordability, the common bean seeds serve as a crucial source of protein, dietary fiber, complex carbohydrates, and other essential nutrients, particularly for low-income populations in Africa and Latin America [2,3]. However, the productivity and quality of common bean crops are significantly threatened by *Colletotrichum lindemuthianum* (Sacc. and Magnus) Briosi and Cavara, a hemibiotrophic ascomycete fungus that causes anthracnose (ANT) [4,5]. This pathogen represents one of the most severe, widespread, and recurring threats to the common bean cultivation. Under favorable environmental conditions, ANT can lead to reduced seed quality and significant yield losses [5,6].

Efforts to control *C. lindemuthianum* have primarily relied on genetic resistance, as the pathogen exhibits high genetic variability that challenges conventional breeding programs [4,5]. Unfortunately, the pathogen has shown a remarkable ability to overcome cultivated plant resistance through coevolution, rendering previously resistant cultivars to become susceptible over time [7,8]. The use of resistant cultivars remains the most effective and environmentally friendly approach for managing *C. lindemuthianum* in common bean cultivation [9]. These cultivars offer a cost-effective and user-friendly solution. However, developing cultivars that are resistant to the diverse range of physiological races of *C. lindemuthianum* poses significant challenges [7].

Anthracnose (ANT) resistance in common beans is conferred by independent loci known as ‘*Co*.’ These resistance loci have been mapped to various chromosomes of the common bean genome, often clustering in disease-resistance regions [10]. Among the identified resistance loci in the Andean genetic pool, many have been mapped to chromosome Pv01. Notable alleles include *Co-1*, *Co-1^2^*, *Co-^3^*, *Co-1^4^*, *Co-1^5^* and *Co-1^HY^* at the *Co-1* locus [11,12,13,14], *Co-x* [15,16], *Co-Pa* [17], *CoPv01^CDRK^* [18], and *Co-AC* [19], all located at the end of Pv01.

The identification and molecular characterization of novel resistance genes plays a crucial role in breeding for disease resistance [20,21]. Gene expression analysis has proven to be a valuable tool in understanding the resistance response of common bean genotypes SEL 1308 and T9576R to the race 73 of *C. lindemuthianum* [22,23]. Several defense-related genes, including those encoding pathogenesis-related proteins (*PR*) such as *PR1a*, *PR1b*, and *PR2*, were found to be spatially and temporally induced by the pathogen [22,23,24]. Notably, a potential gene associated with the *Co-1^2^* locus was identified [23]. Similarly, changes in the expression levels of *PR1a*, *PR1b,* and *PR2* genes were reported during incompatible interactions with race 2 of *C. lindemuthianum* [25]. 

In the evaluation of expression levels of candidate genes for the *Co-1^2^* allele of the *Co-1* gene in response to race 73 of *C. lindemuthianum*, the gene *Phvul.001G243800* exhibited high induction, suggesting its potential as a candidate gene for the *Co-1^2^* allele of the *Co-1* gene [23]. Additionally, through fine mapping in the Hongyundou genotype, four candidate genes were identified for the *Co-1^HY^* allele. Among them, *Phvul.001G243600* and *Phvul.001G243700* showed higher induction in response to race 81 of *C. lindemuthianum*, indicating their potential as candidate genes for the *Co-1^HY^* locus [13]. Moreover, in a genetic study focusing on the anthracnose resistance *Co-x* gene, a gene called *KTR2/3* was found within a CRINKLY4 kinase cluster located between the *Phvul.001G243600* and *Phvul.001G243700* genes. Gene expression analysis revealed that *KTR2/3* was induced by strain 100 (race 3993) of *C. lindemuthianum*, and its transient expression in susceptible genotype BAT93 resulted in increased resistance to the pathogen [16]. The authors suggested that this gene may act as a decoy involved in indirectly recognizing fungal effectors [16]. These findings highlight the significance of gene expression analysis in uncovering potential resistance genes and their roles in the common bean’s defense response to *C. lindemuthianum*.

Through fine mapping, five candidate genes for the *CoPv01^CDRK^* locus, which confer resistance to anthracnose and angular leaf spot in the CDRK common bean cultivar, were identified [18]. Among these candidate genes, gene expression analysis revealed that *Phvul.001G246300*, potentially encoding an Abscisic Acid Receptor (PYL5), exhibited the highest responsiveness to both pathogens, making it the primary candidate gene for the *CoPv01^CDRK^* locus [26]. 

Another noteworthy common bean cultivar, AC, is an Andean landrace collected in the state of Santa Catarina, Brazil [19]. AC possesses the *Co-AC* gene that confers resistance to races 2, 7, 9, 19, 23, 39, 55, 65, 73, 89, 1545, 2047, and 3481 of *C. lindemuthianum* [27]. Through fine mapping, the *Co-AC* gene was located at the end of the Pv01 chromosome within a genomic region containing three candidate genes: *Phvul.001G244300*, *Phvul.001G244400*, and *Phvul.001G244500* [19].

Candidate genes located within the *Co-AC* loci on Pv01 demonstrate unique expression patterns when inoculated with race 73 of *C. lindemuthianum* in the AC cultivar. The primary aim of this study was to investigate the dynamic expression profiles of *Co-AC* candidate genes *Phvul.001G244300*, *Phvul.001G244400*, and *Phvul.001G244500* within the AC cultivar upon exposure to *C. lindemuthianum* race 73. The present study hypothesizes that each of the candidate genes that overlap with the *Co-AC* loci on Pv01 exhibits distinct expression patterns in response to inoculations with race 73 of *C. lindemuthianum* in the AC cultivar. The objective of this study was to investigate the relative expression patterns of the *Co-AC* candidate genes and other disease-resistance genes in the AC cultivar in response to *C. lindemuthianum* race 73, employing quantitative real-time PCR. Specifically, the focus was to gain insights into their potential roles in the plant’s defense mechanisms against this pathogen, contributing to a deeper understanding of disease resistance in common beans.

## 2. Results

### 2.1. Phenotypic Evaluation of the Cultivars 

**Figure 1:**
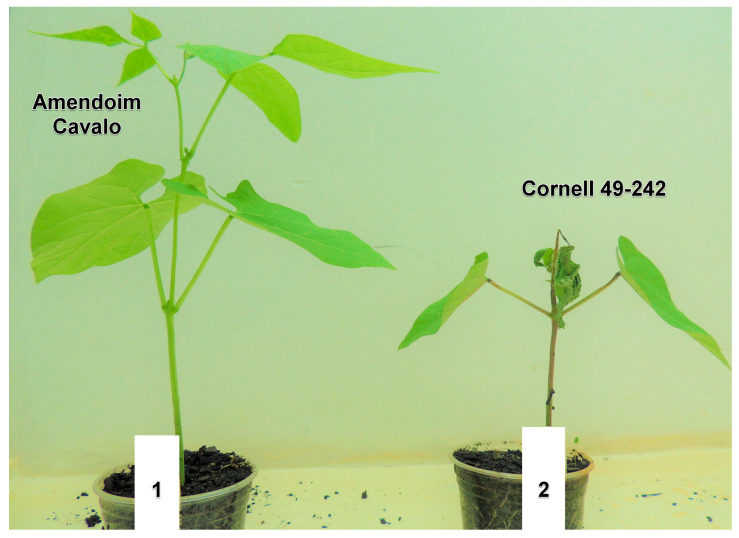
Disease reaction of the resistant cultivar Amendoim Cavalo (**1**) and susceptible cultivar Cornell 49-242 (**2**) after 72 h post-inoculation with race 73 of *Colletotrichum lindemuthianum*.

Inoculation of *C. lindemuthianum* race 73 on the resistant cultivar AC and the susceptible cultivar Cornell 49-242 resulted in disease development exclusively in the susceptible cultivar (Figure 1). Disease symptoms manifested as small water-soaked lesions on the underside of the leaves and small sunken lesions on the stems, ultimately leading to plant mortality. Notably, symptom expression occurred only after 72 h post-inoculation (hpi), indicating the hemibiotrophic nature of the fungus. Conversely, no symptoms or hypersensitive responses were observed in the resistant cultivar.

### 2.2. Differential Expression of Candidate in the Amendoim Cavalo Cultivar Inoculated with Race 73 of C. lindemuthianum

Aiming to identify the molecular mechanisms underlying *Co-AC* resistance, the relative expression of the following candidate genes was assessed: *KTR2/3*, *Phvul.001G243800*, *Phvul.001G244300*, *Phvul.001G244400*, *Phvul.001G244500*, *Phvul.001G245300*, and *Phvul.001G246300*. Additionally, the expression of defense genes *PR1a*, *PR1b*, and *PR2* were evaluated as markers for resistance upon *C. lindemuthianum* race 73 inoculation. These evaluated genes with functional annotation are described in Table S1. 

**Figure 2:**
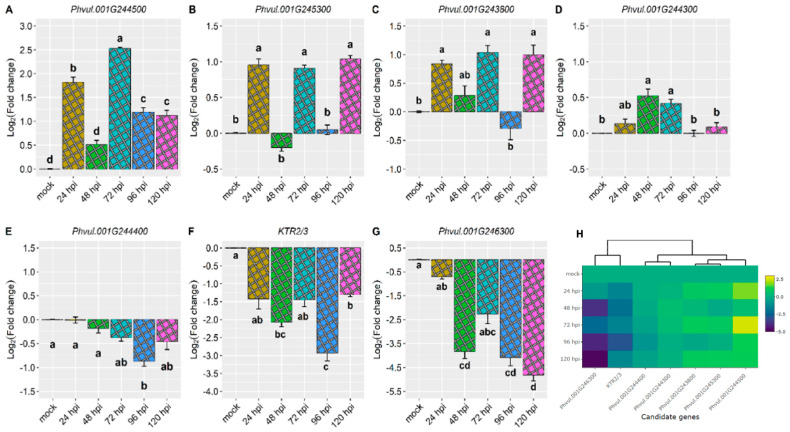
Relative expression of candidate genes (**A**) *Phvul.001G244500,* (**B**) *Phvul.001G245300,* (**C**) *Phvul.001G243800,* (**D**) *Phvul.001G244300,* (**E**) *Phvul.001G2444400,* (**F**) *KTR2/3*, (**G**) *Phvul.001G246300* in the Amendoim Cavalo at 24, 48, 72, 96, and 120 h post-inoculation (hpi) with the race 73 of *C. lindemuthianum* and mock. The results are presented as logarithmic base 2 of the fold change of gene expression. Means with the same letter for each gene are not significantly different at the 5% significance level, using the Alexander–Govern test. (**H**) Heatmap of the relative expression of candidate genes for the *Co-AC* and genes proximal to this *locus* in the Amendoim Cavalo cultivar. Yellow shading indicates higher expression, and dark blue shading has lower expression than reference genes.

*Phvul.001G244500* exhibited the most significant response to the pathogen, showing a 2.5-fold change at 72 h post-inoculation (hpi). Additionally, an approximately 1.8-fold increase was observed at 24 hpi, while increases higher than 1.0-fold were observed at 96 and 120 hpi (Figure 2A,H and Table 1). The gene *Phvul.001G245300* displayed the second-highest response to the pathogen, being induced at 24, 72, and 120 hpi with an average increase of 0.90-fold (Figure 2B,H and Table 1). The gene *Phvul.001G243800* was also induced at 24, 72, and 120 hpi, albeit with a relatively small average increase of 0.9-fold (Figure 2C,H and Table 1).

The expression of the *Phvul.001G244300* gene was induced by the pathogen at 48 and 72 hpi, showing a small increase of 0.4-fold (Figure 2D,H and Table 1). Conversely, the *Phvul.001G244400* gene was downregulated only at 96 hpi, with a reduction of 0.9-fold (Figure 2E,H and Table 1). The *KTR2/3* and *Phvul.001G246300* genes exhibited downregulation at 48, 96, and 120 hpi (Figure 2F–H and Table 1).

The *Phvul.001G244500* gene was the most responsive candidate gene to the pathogen, particularly at 72 hpi. Additionally, the *Phvul.001G246300* candidate gene for *CoPv01^CDRK^* and the *KTR2/3* candidate gene for *Co-x* were significantly downregulated in the AC cultivar upon inoculation with the race 73 of *C. lindemuthianum*. This suggests that different candidate genes are expressed in each anthracnose-resistant cultivar (Figure 2 and Table 1). 

### 2.3. Expression Profile of Defense Genes in the Amendoim Cavalo Cultivar Inoculated with Race 73 of C. lindemuthianum

**Figure 3:**
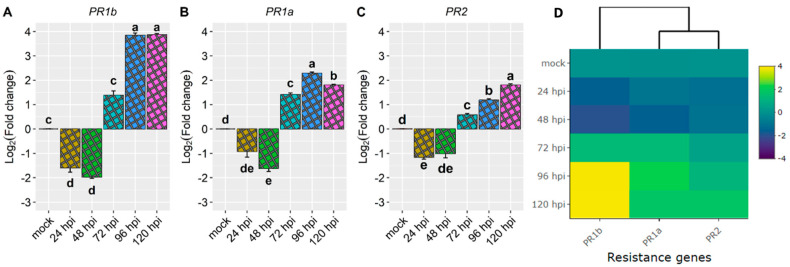
Relative expression of plant defense genes (**A**) *Phvul.006G196900* (*PR1b*), (**B**) *Phvul.003G109100* (*PR1a*)*,* and (**C**) *Phvul.009G256400* (*PR2*) in the common bean cultivar Amendoim Cavalo at 24, 48, 72, 96, and 120 h post-inoculation (hpi) with the race 73 of *C. lindemuthianum* and mock. The results are presented as logarithmic base 2 of the fold change of gene expression. Means with the same letter for each gene are not significantly different at the 5% significance level, using the Alexander–Govern test. (**D**) Heatmap of the relative expression of plant defense genes *Phvul.006G196900* (*PR1b*), *Phvul.003G109100* (*PR1a*), and *Phvul.009G256400* (*PR2*) in the common bean cultivar Amendoim Cavalo Yellow shading indicates higher expression and dark blue shading lower expression than that of reference genes.

Concerning the PR genes, namely *PR1b* (*Phvul.006G196900*), *PR2* (*Phvul.009G256400*), and *PR1a* (*Phvul.003G109100*), their induction was observed only starting from 72 h post-inoculation (hpi), with *PR1b* displaying notable activation between 96 and 120 hpi (refer to Figure 3 and Table 1). These findings suggest that the initiation of the resistance response may be attributed to the candidate gene *Phvul.001G244500*. Furthermore, by 72 hpi, additional defense genes appear to contribute to the activation of the resistance response.

Among them, *PR1b* (*Phvul.006G196900*) stood out as the most responsive to the pathogen, exhibiting a substantial increase in expression from 72 to 96 hpi and a remarkable 3.9-fold increase at 120 hpi (Figure 3A,D and Table 1). The gene *PR1a* (*Phvul.003G109100*) showed a moderate level of expression and response upon exposure to the pathogen. Remarkably, there was a 2.3-fold increase in expression observed at 96 hpi (Figure 3B,D and Table 1). The expression pattern of the *PR2* (*Phvul.009G256400*) gene mirrored that of *PR1a*. It experienced downregulation at 24 and 48 hpi, with a one-fold reduction, but demonstrated increased expression levels at 72, 96, and 120 hpi with fold changes of 0.6, 1.2, and 1.8, respectively (Figure 3C,D and Table 1). 

## 3. Discussion

Gene expression analysis plays a crucial role in understanding the genetic basis of disease resistance and can aid in the identification of effective resistance genes for plant breeding and molecular studies within specific pathosystems. In this study, the resistance response to *C. lindemuthianum* in the AC cultivar was investigated, focusing on the relative expression of candidate genes associated with resistance genes, namely *Co-AC* [19], *CoPv01^CDRK^* [18], *Co-x* [15,16], and the *Co-1^2^* allele for the *Co-1* locus [23] (Figure 2). Additionally, the relative expression of disease-resistance genes *PR1a, PR1b*, and *PR2* was examined in the same pathosystem (Figure 3).

This study focused on a genomic region spanning 250 Kb at the end of Pv01, which encompasses the candidate genes for *Co-AC* and potential genes closely linked to this locus (Figure S1). Notably, distinct expression patterns among the candidate genes of the *Co-AC* resistance gene in the AC common bean cultivar were observed. Among these candidate genes (*Phvul.001G244300*, *Phvul.001G244400*, and *Phvul.001G244500*), *Phvul.001G244500* displayed the most pronounced responsiveness to race 73 of *C. lindemuthianum*, particularly at 72 hpi, with a 2.5-fold change in gene expression (Figure 2 and Figure 3 and Table 1).

These findings highlight the potential role of the *Phvul.001G244500* gene, which encodes a Basic Helix–Loop–Helix (bHLH) transcription factor, in regulating defense processes against *C. lindemuthianum* race 73. Proteins containing the Basic Helix–Loop–Helix domain are known to regulate the expression of their target genes, which are involved in many physiological processes and have a broad range of functions in biosynthesis, metabolism, and transduction of plant hormones [28]. Newly, bHLHs were observed differentially expressed in rose petals upon disease infection, suggesting candidate genes that regulate the response of rose plants to *Botrytis cinerea* [29]. 

Unlike the robust induction of *Phvul.001G244500* in the AC cultivar, a previous study showed a low expression pattern of this candidate gene in the California Dark Red Kidney (CDRK) cultivar inoculated with *C. lindemuthianum* race 73. Lovatto [26] et al. (2023) reported that *Phvul.001G244500* displayed only a slight induction at 120 hpi, with less than a one-fold change in gene expression. *Phvul.001G244500* is a strong candidate for the *Co-AC* resistance gene in the AC cultivar, while it was not responsive in the CDRK cultivar.

*Phvul.001G245300* expression in AC cultivar was the second most induced gene, although at lower levels, with approximately 1.0-fold change at 24, 72, and 120 hpi. This observation suggests that *Phvul.001G245300* may function in a secondary layer of the resistance response. *Phvul.001G245300* encodes a putative Leucine-Rich Repeat Protein Kinase (LRR-Kinase), and proteins encoding LRR and Kinase domains are known to be expressed by resistance genes [10].

The AC cultivar *Phvul.001G243800* showed only minor increases in expression levels at 24, 72, and 120 hpi (approximately one-fold change). In contrast, the *Phvul.001G244500* gene exhibited a substantial 2.5-fold change in gene expression (Figure 2 and Figure 3 and Table 1). These results suggest that *Phvul.001G243800* has a limited effect on the resistance response in the AC cultivar. The *Phvul.001G243800* gene, which is a candidate for the *Co-1^2^* allele of the *Co-1* locus, was found to be highly induced at 72 hpi in the T9576R genotype inoculated with race 73 of *C. lindemuthianum* [23]. Therefore, besides the allelism test, this candidate gene expression study corroborates that different genes confer resistance to the same pathogen in each cultivar once each resistant cultivar expresses high levels of different candidate genes. 

The present study demonstrated a different pattern for the *KTR2/3* gene in the AC cultivar inoculated with race 73 of *C. lindemuthianum*. It was consistently downregulated at 48, 96, and 120 hpi, indicating that this gene may not trigger the resistance response in this specific pathosystem. Conversely, the *KTR2/3*, a candidate gene for the *Co-x* gene, was upregulated at 24 hpi in the JaloEEP558 cultivar [16]. Again, this corroborates that different genes confer resistance to the same pathogen in each cultivar.

In this study, the *Phvul.001G246300* gene in the AC cultivar was downregulated, suggesting that it may not be the responsive resistance gene in this specific pathosystem. On the other hand, in the CDRK cultivar, the *Phvul.001G246300* gene showed the highest responsiveness to both *C. lindemuthianum* race 73 and *P. griseola* race 63-39, indicating that the *CoPv01^CDRK^* resistance gene confers resistance to both diseases in common bean. Therefore, the expression levels of candidate genes of both resistant cultivars indicate that different genetic resistances are involved in each cultivar.

Taken together, it is possible to identify distinct roles of the genes *Phvul.001G244500*, *Phvul.001G243800*, *KTR2/3*, and *Phvul.001G246300* involved in the resistance response to race 73 of *C. lindemuthianum* in different common bean cultivars. The contrasting expression patterns emphasize the complexity of resistance mechanisms and highlight the importance of the candidate gene *Phvul.001G244500* for *Co-AC* in conferring robust resistance. 

PR proteins are induced by phytopathogens as well as defense-related signaling molecules. They are the key components of the plant’s innate immune system, especially systemic acquired resistance (SAR), and are widely used as diagnostic molecular markers of defense signaling pathways [30]. Plant resistance to pathogens involves the activation of genes encoding *PR* proteins, which are categorized into 17 families and are known to accumulate following pathogen infection in various plant species [31]. *PR1* genes, a subset of the PR family, are commonly used as markers for systemic acquired resistance [32]. However, the understanding of *PR1* genes remains limited, with only a small proportion having been studied thus far [33].

In this current investigation, it was noted that among the tested defense resistance genes, *PR1b* displayed the highest level of responsiveness to race 73 of *C. lindemuthianum* in the AC cultivar, particularly evident at 96 and 120 hpi (refer to Figure 3). Interestingly, *PR1b* also exhibited the most conspicuous induction in the CDRK cultivar upon inoculation with race 73 of *C. lindemuthianum*, predominantly at 120 hpi [25].

Theoretical considerations propose that *PR1b* encodes a *PR1*-like protein, typically secreted into the extracellular spaces of plant leaves, as a response to pathogen infection [34]. In *Arabidopsis thaliana*, a homolog of *PR1b* plays a crucial role in defense responses against necrotrophic pathogens, mediated by methyl jasmonate and ethylene while being repressed by salicylic acid [35]. 

In this investigation, *PR1a* demonstrated significant upregulation in the AC cultivar when inoculated with race 73 of *C. lindemuthianum*, particularly peaking between 72 and 120 hpi, with a notable zenith at 96 hpi (refer to Figure 3 and Table 1). Likewise, in the CDRK cultivar subjected to the same inoculation, *PR1a* exhibited its highest induction at 72 and 96 hpi [26]. The upregulation of *PR1a* was also evident in the SEL 1308 cultivar following inoculation with race 73 of *C. lindemuthianum* [22,24], as well as in the T9576R common bean when exposed to race 73 of *C. lindemuthianum* [23], and in the ‘Naz’ cultivar during inoculation with race 2 of *C. lindemuthianum* [25].

PR1a is postulated to encode a PR protein featuring the Bet v I domain [36]. A recent transcriptome study, delving into the incompatible interaction between strain C531 and the BAT93 cultivar, underscored the pivotal role of PR10/Bet v I in conferring disease resistance in common beans [37].

In this study involving the AC cultivar inoculated with race 73 of *C. lindemuthianum*, the expression of *PR2* exhibited a modest repression at 24 hpi, followed by a substantial induction from 72 hpi, reaching a notable peak at 120 hpi. Similarly, in the CDRK cultivar, *PR2* displayed an upregulation in response to race 73 of *C. lindemuthianum*, particularly between 72 and 96 hpi [26]. Examining the SEL 1308 cultivar, *PR2* was also observed to be upregulated following inoculation with race 73 of *C. lindemuthianum* [22,24]. In the case of the Naz cultivar, known for its resistance to race 2 of *C. lindemuthianum*, an upregulation of PR2 was noted from 48 hpi onwards [25]. In the T9576R genotype subjected to inoculation with race 73 of *C. lindemuthianum*, *PR2* exhibited upregulation at all evaluated time points except at 96 h post-inoculation [23].

Hypothetically, it is suggested that *PR2* encodes a 1,3-beta-glucan endohydrolase [38]. Consequently, PR2 may play a pivotal role in the resistance response by actively degrading fungal cell walls, potentially triggering a plant’s pattern-triggered immunity (PTI) [39,40].

Gene expression analysis offers valuable insights into the role and interaction of these genes in mounting an effective resistance response. Moreover, it provides additional knowledge necessary for the identification of promising genes for utilization in plant breeding programs. The most expressed candidate genes can be validated by phenotypic evaluation of the same cultivar with candidate gene knockout and ultimately developing molecular markers for enhanced selection and incorporation of effective genes into breeding strategies.

It is crucial to emphasize that the Andean AC cultivar is resistant to 13 out of the 15 *Colletotrichum lindemuthianum* races assessed [19]. Additionally, this cultivar exhibits commendable agronomic traits. Previous studies conducted by Vidigal Filho and colleagues (2020) [3] demonstrated that the same cultivar displayed a broad spectrum of resistance, covering four distinct races of *C. lindemuthianum*.

These findings contribute valuable knowledge regarding the genetic mechanisms underlying resistance to *C. lindemuthianum* in the AC cultivar and provide insights into potential genes involved in the resistance response to anthracnose in common beans. These results have implications for future research and can aid in the development of effective strategies for anthracnose resistance in common bean breeding programs. The characterization of a specific candidate gene, *Phvul.001G244500*, broadens the understanding of the defense networks activated in response to pathogen infection. Additionally, *PR1a*, *PR1b,* and *PR2* would play significant roles in the defense responses of different common bean cultivars against *C. lindemuthianum*. 

## 4. Conclusions

The observed upregulation of candidate genes in the incompatible interaction may signify the host’s response to counterbalance the compromised resistance caused by pathogen infection. This research has successfully pinpointed the candidate gene *Phvul.001G244500* as an effective defense mechanism against the specific race 73 of *C. lindemuthianum*. Moreover, the involvement of defense genes *PR1a*, *PR1b,* and *PR2* in the resistance response was demonstrated, with a particular emphasis on *PR1b*. This study has yielded invaluable insights into the genetic underpinnings of resistance to *C. lindemuthianum* race 73 within the AC cultivar. These discoveries significantly bolster our capacity to develop more effective strategies for breeding anthracnose-resistant common bean cultivars. By incorporating this resistance gene into breeding programs, these findings can enhance the resilience of common bean crops against this devastating pathogen, contributing to sustainable agriculture and food security.

## 5. Materials and Methods

### 5.1. Plant Material and Growth Conditions

The experiment was performed in a completely randomized design. Seedlings of the resistant AC and the susceptive Cornell 49-242 cultivars were inoculated with race 73 of *C. lindemuthianum*, and relative expression of ten genes only in AC cultivar was evaluated at 24, 48, 72, 96, and 120 hpi and in the mock. Three biological replicates (plants) were collected for each experimental condition evaluated, and for each biological replicate, three technical replicates (qPCR reactions) were performed in each experiment. The experiment was conducted at the Núcleo de Pesquisa Aplicada à Agricultura (Nupagri) at the Universidade Estadual de Maringá (UEM) in Maringá, Paraná, Brazil (latitude 23° 26′8″ S, longitude 51° 53′42″ W). Briefly, seeds were planted in plastic trays filled with a commercial substrate, MecPlant (MEC PREC—Ind. Com Ltd., Telemaco Borba, Brazil), that had been previously sterilized and fertilized. The seedlings were grown in greenhouses under natural light at a temperature of 25 °C until the first trifoliate leaf growth stage [8].

### 5.2. Pathogenesis Assay

Monosporic cultures of *C. lindemuthianum* were prepared following the methodologies described by Mathur et al. [41]. The inoculum was produced on a medium comprising green common bean pods incubated at 22 ± 2 °C without light for 14 days. Conidiospore quantification was conducted using a hemacytometer under an optical microscope. The plants were sprayed with a conidiospore suspension prepared in distilled water and Tween 20^®^ (0.01%) at an approximate concentration of 1.2 × 10^6^ mL^−1^. Inoculation was carried out by spraying the suspension onto the plants using a manual pressurized pump sprayer. For the negative control (mock), plants were sprayed only with distilled water and Tween 20^®^ (0.01%). After inoculation, the plants were placed in a mist chamber at a temperature of 22 ± 2 °C, a photoperiod of 12 h, and ≥95% relative humidity for 72 h. Subsequently, the plants were transferred to a growth chamber with a temperature of 22 ± 2 °C and a photoperiod of 12 h for the duration of the experiment. Anthracnose symptoms were evaluated using the 1-to-9 disease severity scales proposed by Pastor-Corrales et al. [8]. Plants with disease reaction scores between 1 and 3 were considered resistant, whereas plants with scores from 4 to 9 were considered susceptible.

### 5.3. RNA Extraction 

For sample collection and total RNA extraction, leaf samples weighing approximately 100 ± 10 mg from the AC cultivar inoculated with race 73 of *C. lindemuthianum* were collected at 24, 48, 72, 96, and 120 h post-inoculation (hpi), as well as from the mock control. The leaf samples were immediately submerged in liquid nitrogen (N_2_) and stored at −80 °C until further processing. Total RNA extraction was performed by macerating the tissue and adding 1000 µL of TRIzol^®^ (Invitrogen™, Waltham, MA, USA) to each microtube. The subsequent steps for RNA extraction and isolation followed the manufacturer’s recommendations. The precipitated total RNA pellets were washed with 70% ethanol (EtOH) and then suspended in RNase-free H_2_O.

The integrity of the total RNA was assessed by electrophoresis on a 1% m/v agarose gel, run for 80 min at 80 volts, at 5 °C, and in the absence of light. To assess the quality and quantity of total RNA, a spectrophotometer (FEMTO 700STM) was used to measure absorbance at 230 nm, 240 nm, 260 nm, and 280 nm. The following absorbance ratios were used to determine RNA purity: A_260_/A_230_ between 1.9 and 2.4, A_260_/A_240_ ≥ 1.4, and A_260_/A_280_ between 1.8 and 2.2. The concentration of total RNA was calculated using the formula [RNA] (ng µL^−1^) = A_260_nm × 40 × 100 [42]. Total RNA samples that met the purity criteria and exhibited no visual signs of degradation were subjected to DNase I treatment using DNase ITM (Invitrogen™, Waltham, MA, USA) to remove any residual DNA. The purification reaction was carried out using 1 µg of total RNA, following the manufacturer’s instructions.

### 5.4. Reverse Transcription (cDNA Synthesis)

The cDNA synthesis was carried out using the ‘Superscript^®^ IV First-Strand Synthesis System’ kit (Invitrogen™, Waltham, MA, USA) following the manufacturer’s protocol. The total volume of cDNA synthesis reaction was 20 µL with the following components: 1 µg of total RNA, primer-oligo d(T) (2.5 µM), dNTP mix (0.5 mM each), First-Strand Buffer (1X), DL-dithiothreitol (5 mM), ribonuclease inhibitor (2 U µL^−1^), MMLV-RT (10 U µL^−1^) and RNase-free water. Initially, total RNA, primer-oligo d(T), dNTP mix, and RNase-free water (to 13 µL) were added to the reaction. The samples were incubated in a thermocycler (Applied Biosystems^®^ Veriti^®^ 96-Well Fast Thermal Cycler, Waltham, MA, USA) at 65 °C for 5 min, followed by 4 °C for 1 min. Then, the First-Strand Buffer, DL-dithiothreitol, ribonuclease inhibitor, and 1 MMLV-RT were added to the reaction. The samples were incubated at 55 °C for 10 min for cDNA synthesis activation, followed by 80 °C for 10 min to inactivate the reaction. To remove residual RNA after cDNA synthesis, 1 µL of *Escherichia coli* RNase H was added, and the samples were incubated at 37 °C for 20 min. The cDNA synthesis product (20 µL) was diluted 1:100 for qPCR analysis. To assess the cDNA synthesis efficiency, positive control was included, in which HeLa-S3 RNA (10 ng) was used instead of total RNA.

For the control of cDNA synthesis, the PCR reaction was conducted using the following components: 5 µL of PCR buffer (10X), 2 µL of MgCl_2_ (50 mM), 1 µL of dNTP Mix (10 mM), 1 µL of sense primer (10 µM), 1 µL of antisense primer (10 µM), 2 µL of cDNA for the positive control, and 2 µL of ultrapure H_2_O for the negative control. Additionally, 0.2 µL of Taq Platinum^TM^ DNA polymerase (Invitrogen™, Waltham, MA, USA) and 37.8 µL of ultrapure H_2_O were included in the reaction mixture. The PCR amplification was performed under the following thermocycling conditions: an initial denaturation step at 94 °C for 2 min, followed by 35 cycles of denaturation at 94 °C for 15 s, annealing at 55 °C for 30 s, and synthesis at 68 °C for 1 min. After completion of the PCR reaction, both the positive and negative controls were subjected to electrophoretic analysis using a 1.5% (*w*/*v*) agarose gel. The expected fragment size of approximately 353 bp was observed in the positive control lane, confirming the successful amplification, while no fragment was detected in the negative control lane, validating the absence of non-specific amplification as indicated by the manufacturer’s instructions.

### 5.5. Target Genes and Primer Design

The candidate genes selected for expression analysis in the AC cultivar were associated with resistance to race 73 of *C. lindemuthianum*. Specifically, the genes *Phvul.001G244300*, *Phvul.001G244400*, and *Phvul.001G244500* were identified within the *Co-AC* locus of the AC cultivar [19]. Additionally, the gene *Phvul.001G246300*, located within the *CoPv01^CDRK^* loci, exhibited significant responsiveness in the CDRK cultivar when inoculated with race 73 of *C. lindemuthianum* [26]. The gene *Phvul.001G245300* is located in close proximity to the genomic region of the CDRK cultivar. The inclusion of the *Phvul.001G243800* gene was based on its induction in the near-isogenic line T9576R, possessing the *Co-1^2^* resistance allele, when inoculated with race 73 of *C. lindemuthianum* [23]. The *KTR2/3* candidate gene for *Co-x* in the Jalo EEP558 cultivar was also evaluated due to its induction in response to race 3993 of *C. lindemuthianum* [16]. Furthermore, the well-known plant defense genes *Phvul.003G109100* (*PR1a*), *Phvul.006G196900* (*PR1b*), and *Phvul.009G256400* (*PR2*) were included in the analysis [22,23,24]. To standardize gene expression levels, the reference genes *Phvul.008G011000* (actin—ACT) and *Phvul.001G133200* (insulin-degrading enzyme—IDE) [43] were used. ACT had previously been validated for quantifying the relative expression of candidate genes in studies [23,25], while IDE’s validation was previously established by Oblessuc et al. [24]. Both genes have been utilized as reference genes for quantifying the relative expression of resistance genes against ANT in studies [16,43]. For normalization purposes, the reference genes *Phvul.008G011000* (ACT) and *Phvul.001G133200* (IDE) were used [43].

To obtain the coding sequences (CDS) and DNA sequences of the target genes, the common bean (*P. vulgaris* L.) genome available at v1.2 Phytozome [44] was accessed. Primer design for the qPCR assay was performed using the ‘Primer-Blast web tool’ [45] with the following specifications: primer size between 18 and 24 base pairs (bp), melting temperature between 59 and 61 °C, amplicon size between 80 and 160 bp, and, when possible, the primer pair should be separated by at least one intron in the corresponding genomic DNA sequence. Primer dimers and secondary structures were assessed using Gene Runner software (version 6.5.52), the ‘Multiple Prime Analyzer’ web tool (Thermo Fisher Scientific, Waltham, MA, USA): https://bit.ly/34kZpnP, accessed on 11 May 2020), and ‘The Sequence Manipulation Suite’ web tool [46]. The secondary structure of the amplicons was verified using ‘The Mfold Web Server’ platform [47] with coding sequences obtained from v1.2 Phytozome. All primer designs and in silico validation procedures not explicitly mentioned followed established literature recommendations [48,49]. Table S2 provides the primer sequences for each candidate gene evaluated, with the primers for the *KTR2/3* gene obtained from Richard et al. [16].

### 5.6. Quantitative PCR (qPCR) and Data Analysis

The determination of PCR efficiency for each primer involved establishing a standard curve through a fivefold serial dilution, utilizing the cDNA pool as the template. This process incorporated three replicates at every dilution point [50,51]. The amplification efficiency was computed employing the equation E = [10^(−1/slope^)] − 1 [51], using the slope values derived from linear regression analysis. This analysis encompassed the log_10_-transformed cDNA concentrations on the x-axis and corresponding Cq values on the y-axis. The calculated amplification efficiency for each primer pair ranged from 0.92 to 1.09 while maintaining a coefficient of determination (R^2^) for the linear regression of at least 0.98 (Table S2).

The cDNA quantification reactions were conducted in the StepOnePlus™ real-time PCR system (Applied Biosystems™, Waltham, MA, USA; StepOnePlus™ Real-Time PCR Systems) using 96-well microplates [MicroAmp™ Fast 96-well Reaction Plate (0.1 mL)] sealed with MicroAmp™ Optical Adhesive Film. The total reaction volume was 10 µL, consisting of 3.4 µL of cDNA, 1.6 µL of forward and reverse primer mix (800 nM), and 5 µL of PowerUp™ SYBR™ Green Master Mix (Applied Biosystems™, Waltham, MA, USA). The thermocycling conditions included 50 °C for 2 min, 95 °C for 2 min, 40 cycles of 15 s at 95 °C, and 30 s at 60 °C. 

After completing the cDNA quantitation reaction, a thorough assessment of target specificity was conducted through a dissociation curve analysis, employing the continuous melt curve setup as per the manufacturer’s specifications. Only samples demonstrating clear specificity in accordance with the dissociation curve were considered for subsequent analysis. Quantification cycle (Cq) values were extracted using StepOnePlus™ Software v2.3 (Applied Biosystems™, Waltham, MA, USA). The baseline was automatically established, and the threshold was set manually during the exponential phase of amplification. For all cDNA quantification reactions, a consistent threshold value of 0.7707 was applied.”

The genes *Phvul.008G011000* (IDE) and *Phvul.001G133200* (ACT) served as reference genes [43]. The arithmetic mean of quantification cycle (Cq) values [52] was computed for each experimental condition under consideration. Relative expression levels were determined by normalizing Cq values with the reference genes, employing the 2^−ΔΔCT^ method [53,54]. Mean Cq values were derived for each gene in every experimental condition through the calculation based on three biological repetitions and three replicates (n = 3 × 3). 

The investigation into the relative expression of candidate genes at the Co-AC locus and known disease-resistance genes was conducted in response to race 73 of *C. lindemuthianum*, spanning time points at 24, 48, 72, 96, and 120 h post-inoculation (hpi) in the AC cultivar. The calibrator condition for each gene was the relative expression observed in the mock (control, without pathogen). For data analysis and presentation of results, a logarithmic base 2 transformation was applied before statistical analysis. The Alexander-Govern test, with a significance level of 5%, was utilized to compare expression levels among experimental conditions. Pairwise comparisons of relative expression means at different time points for each gene were assessed, with significance levels adjusted using Bonferroni correction (*p* ≤ 0.05). These statistical analyses employed the ‘oneway.test’ [55] and ‘companion’ R packages.

All data and statistical analyses were conducted using R software (version 4.0.3) (R Core Team, Vienna, Austria, accessed on 14 June 2021), and plots were generated using the ggplot2 package [56] and R base. Error bars, representing the standard deviation of the means from three biological and three technical replicates (3 × 3), were incorporated into the visualizations. Heatmaps representing mean Cq values were generated using the ‘heatmaply’ R package, and the dendrogram was constructed based on the Euclidean distance measure and the average linkage function [50] among the relative expression values of the genes.

## Figures and Tables

**Table 1:** Summary table of mean relative gene expression (Log2(fold change)) of *Co-AC* candidate genes and pathogenesis-related genes in response to *C. lindemuthianum* race 73 in Amendoim Cavalo cultivar.

Gene	Gene Model	*C. lindemuthianum* Race 73				
		24 hpi	48 hpi	72 hpi	96 hpi	120 hpi
*Co-x*	*KTR2/3*	−1.4	−2.1	−1.4	−2.9	−1.3
*Co-1*	*Phvul.001G243800*	0.8	0.3	1.0	−0.3	1.0
*Co-AC*	*Phvul.001G244300*	0.1	0.5	0.4	0.0	0.1
	*Phvul.001G244400*	0.0	−0.2	−0.4	−0.9	−0.5
	*Phvul.001G244500*	1.8	0.5	2.5	1.2	1.1
*CoPv01^CDRK^*	*Phvul.001G245300*	1.0	−0.2	0.9	0.0	1.0
	*Phvul.001G246300*	−0.7	−3.8	−2.3	−4.1	−4.8
Pathogenesis-related genes	*Phvul.003G109100* (*PR1a*)	−0.9	−1.6	1.4	2.3	1.8
	*Phvul.006G196900* (*PR1b*)	−1.6	−2.0	1.4	3.8	3.9
	*Phvul.009G256400* (*PR2*)	−1.2	−1.0	0.6	1.2	1.8

## Data Availability

All data are presented within the article or in the Supplementary Materials.

## Supplementary Materials

The following supporting information can be downloaded at https://www.mdpi.com/article/10.3390/plants13091245/s1; Table S1: Gene model and predicted functional annotation based on Phytozome; Table S2 Target genes, primers used, qPCR product size (amplicon), primer melting temperature (Tm), amplification efficiency (E) and coefficient of determination of linear regression (R²); Figure S1 Common bean chromosome Pv01 containing candidate genes for anthracnose resistance genes *Co-x* (*KTR2/3*), *Co-1* (*Phvul.001G243800), Co-AC* (*Phvul.001G244300, Phvul.001G244400*, and *Phvul.001G244500*), and *CoPv01^CDRK^* (*Phvul.001G245300* and *Phvul.001G246300*).
