# Supplementary material for: Alterations in Gene Expression during Incompatible Interaction between Amendoim Cavalo Common Bean and Colletotrichum lindemuthianum
Source: Plants (Basel). 2024 Apr 30;13(9):1245. doi: 10.3390/plants13091245 (PMC11085365; doi:10.3390/plants13091245)
Supplement: Supplementary file 1 [file plants-13-01245-s001.zip › plants-2788738-supplementary.pdf]

Table S1 Gene model and predicted functional annotation based on Phytozome.

Gene model	Gene	Predicted functional annotation on Phytozome
KTR2/3	*Co-x*	*Serine/Threonine-protein kinase-like protein CCR3- related*
Phvul.001G243800	*Co-1*	Serine/Threonine-Protein Kinase-Like Protein CCR3-Related
Phvul.001G244300	*Co-AC*	Clathrin Heavy Chain (CLTC)
Phvul.001G244400	*Co-AC*	Uncharacterized protein
Phvul.001G244500	*Co-AC*	Basic Helix-Loop-Helix (bHLH) domain-containing protein with possible regulation of transcription function
Phvul.001G245300	*CoPv01^{CDRK}*	Protein Tyrosine Kinase (Pkinase_Tyr) // Leucine Rich Repeat N-terminal domain (LRRNT_2)
Phvul.001G246300	*CoPv01^{CDRK}*	Abscisic Acid Receptor PYL5
Phvul.003G109100	*PR1a*	Pathogenesis-related protein 1 (PR1)
Phvul.006G196900	*PR1b*	Pathogenesis-related protein Bet v I family (Bet_v_1))
Phvul.009G256400	*PR2*	Glucan endo-1,3-beta-D-glucosidase / Laminarinase

Table S2 Target genes, primers used, qPCR product size (amplicon), primer melting temperature (Tm), amplification efficiency (E) and coefficient of determination of linear regression (R^2).

Gene Model[a]	Genes	Primers Forward (F) and Reverse (R) (5`-3`)	Tm (°C)	Amplicon (bp)	E[b]	R^{2c}
Phvul.001G133200[*]	*IDE*	F: AAGCAGGTATCTTGGCCATCTC<br>R: AAAGCAAACTCCAAGCTCCAATC	F: 60.16<br>R: 59.99	126	0.92	0.99
Phvul.008G011000[*]	*ACT*	F: ACAGCCAGGACCAGTTCATC<br>R: TGTATGTGGTCTCGTGAATGC	F: 59.67<br>R: 58.38	154	0.93	0.98
Phvul.001G243800	*Co-1*	F: CCTCAAGGTGGGGCTTTTGAG<br>R: TCACCGAGAAACTCCCATTGC	F: 61.16<br>R: 60.61	118	1.01	0.99
KTR2/3	*Co-x*	F: ATGCACAGGGGAATGGGATG<br>R: GCCATAGCGAGTGAGAGTGCG	F: 60.11<br>R: 63.42	279	1.06	0.98
Phvul.001G244300	*Co-AC*	F: GAAACGTCTCCGCAGAATAGTG<br>R: GTCTTGTGTTGTTCCTTGGAGTTG	F: 59.40<br>R: 60.44	150	0.99	0.99
Phvul.001G244400	*Co-AC*	F: TACAGCAAGAGAGCGGTTAAAGG<br>R: CCCTTTGTCACTTTGTTTTGAAGC	F: 60.62<br>R: 59.67	121	1.07	0.99
Phvul.001G244500	*Co-AC*	F: CAATGCACAGCTCGCAACTC<br>R: GGAACTGTGAAAGCTCTGCTAAC	F: 60.45<br>R: 59.81	141	1.09	0.98
Phvul.001G245300	*CoPv01^{CDRK}*	F: TCTGCTGGAAGGGTGGTAGTC<br>R: GGACGTTATGTGAACAAGGTTTGC	F: 61.17<br>R: 61.08	93	1.04	0.99

Phvul.001G246300	*CoPv01[CDRK]*	F: CTTCTTCCCTTCACTTCGATACC	F: 58.57	87	0.95	0.99
		R: GTTGAGAGTGTTTGTGGCAGT	R: 58.98			
Phvul.003G109100	*PR1a*	F: GTCCTAACGGAGGATCACTCA	F: 58.62	148	1.01	0.98
		R: CAGGGATTGGCCAGAAGGTAT	R: 59.50			
Phvul.006G196900	*PR1b*	F: GGTTTGCCTATGATCCCAATGC	F: 59.96	115	0.99	0.99
		R: TGTTGTGAGCGTTGAGGAAGTC	R: 61.06			
Phvul.009G256400	*PR2*	F: CAGAGGTTCTCATTTGCTGCTTTC	F: 60.62	98	1.09	0.99
		R: ATGCCATAACACACCCCGATTTG	R: 61.75			

[a] Based on the *Phaseolus vulgaris* genome available on the Phytozome v1.2 platform: https://phytozome.jgi.doe.gov/pz/portal.html#; * Reference genes;
[b] Amplification efficiency obtained from the Equation $E=[10^{(-1/slope)}]-1$ (Rasmussen, 2001);
[c] Coefficient of determination of linear regression.

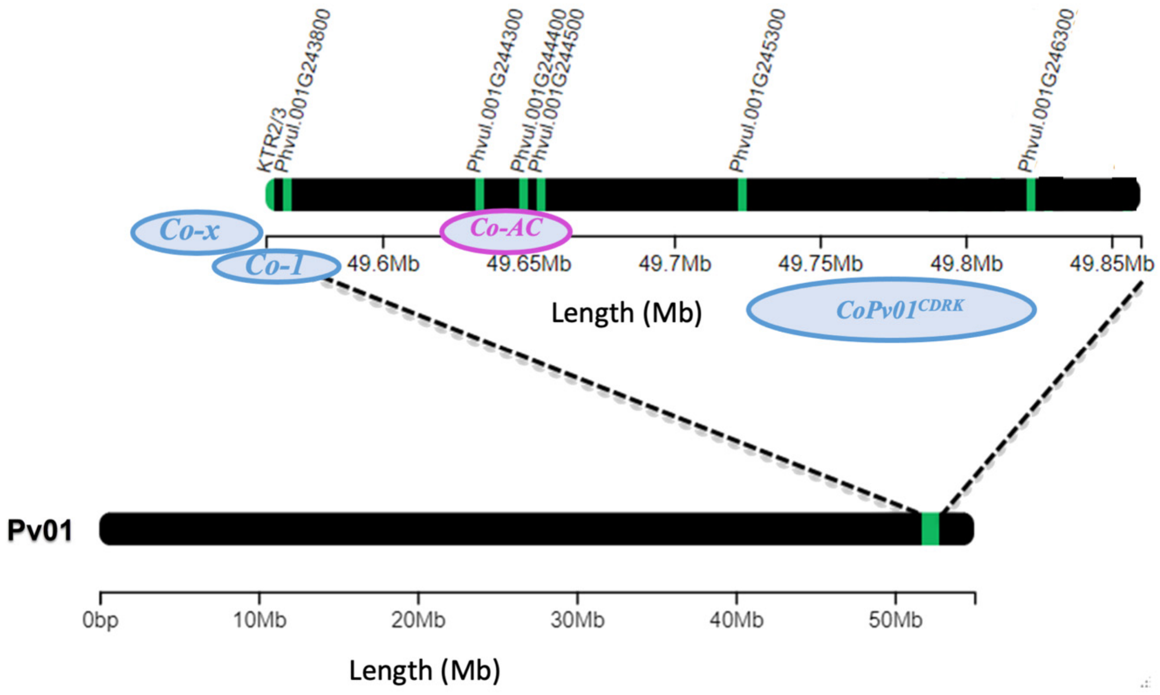

Figure S1 Common bean chromosome Pv01 containing candidate genes for anthracnose resistance genes *Co-x* (*KTR2/3*), *Co-1* (*Phvul.001G243800*), *Co-AC* (*Phvul.001G244300*, *Phvul.001G244400*, and *Phvul.001G244500*), and *CoPv01[CDRK]*/*PhgPv01[CDRK]* (*Phvul.001G245300* and *Phvul.001G246300*).
